# Physical activity, obesity and risk of atherosclerotic cardiovascular diseases among patients with hypertension and diabetes attending a teaching hospital in Edo State, Nigeria

**DOI:** 10.1371/journal.pone.0307526

**Published:** 2024-08-23

**Authors:** Tijani Idris Ahmad Oseni, Sulaiman Dazumi Ahmed, Pauline Etuajie Eromon, Neba Francis Fuh, Isaac Newton Omoregbe

**Affiliations:** 1 Department of Family Medicine, Edo State University, Uzairue, Nigeria; 2 Department of Internal Medicine, Irrua Specialist Teaching Hospital, Irrua, Nigeria; 3 Department of Family Medicine, Irrua Specialist Teaching Hospital, Irrua, Nigeria; 4 Department of Community Medicine, Irrua Specialist Teaching Hospital, Irrua, Nigeria; Delta State University, NIGERIA

## Abstract

**Introduction:**

Preventing Atherosclerotic Cardiovascular Diseases (ASCVD) can best be achieved by promoting a healthy lifestyle through improvements in diet, physical activity, and avoidance of tobacco use and exposure to second-hand smoke. The study aimed to determine the association between physical activity as well as obesity and the risk of atherosclerotic cardiovascular diseases among patients with hypertension and diabetes attending Irrua Specialist Teaching Hospital (ISTH), Irrua, Nigeria.

**Methodology:**

The research was a descriptive, cross-sectional study of 394 systematically selected consenting patients with hypertension and diabetes presenting to a teaching hospital in Irrua, Edo State, Nigeria. The Cardiovascular risk assessment was determined using the Framingham 10year Risk of General Cardiovascular Disease. Anthropometric assessment, blood pressure and blood glucose were determined. Data was collected with a semi-structured questionnaire and analysed with Stata version 16. Chi square and logistic regression was used to test for association and significance level was set at p = 0.05.

**Results:**

The study included 394 participants with a mean age of 54±15.47years. Respondents were mostly females (55.3%), physically inactive (70.3%), overweight (42.4%) and had a high risk (41.8%) of developing CVD in 10 years using Framingham categorisation. There was a significant association between physical activity (P<0.01; OR 2.45; CI: 1.53–3.92), obesity (P<0.01; OR 2.52; CI: 1.64–3.86) and risk of developing CVD.

**Conclusion:**

The study found a statistically significant relationship between physical inactivity, obesity, and the risk of atherosclerotic cardiovascular diseases. Increasing physical activity levels need to be a top priority at all levels of healthcare as well as the general population.

## Introduction

### Background

Cardiovascular diseases (CVDs) are the leading cause of death worldwide caused mainly by atherosclerosis, a chronic inflammatory disease of the blood vessels that result in their narrowing [1, 2]. CVDs which have been found to be on the increase over the past 20 years in Nigeria include hypertension, heart failure, and stroke [1–5].

Over 82 million Americans have one or more forms of cardiovascular diseases (CVD), accounting for 32.8% of all deaths in the United States [4]. Sub-Saharan Africa (SSA) is experiencing an epidemic of cardiovascular diseases (CVDs) on an unimaginable scale [5]. Disability and mortality attributable to CVDs and the traditional risk factors, including hypertension, obesity, diabetes mellitus, and dyslipidaemia, continue to rise in several SSA countries [5]. Adebayo et al reported a prevalence of 37% of cardiovascular disease in a south western state among female patients [6]. A marked increase in the prevalence of CVD of 150% was also reported by Adedapo [6], in South-West Nigeria, and this finding agrees with that of a study by Eze et al on the cardiovascular risk factors in south-eastern Nigeria [7].

Hypertension is the leading risk factor for atherosclerotic cardiovascular diseases [8]. Other risk factors associated with atherosclerosis and cardiovascular diseases include physical inactivity, alcohol consumption, cigarette smoking, overweight and obesity [9]. Thus, preventing ASCVD can best be achieved by promoting a healthy lifestyle throughout life. Prevention strategies must include a strong focus on lifestyle optimization (improvements in diet, physical activity, and avoidance of tobacco use and exposure to second-hand smoke) to minimize the risk of future ASCVD events.

The aim of the study was to determine the association between physical activity and obesity and the risk of atherosclerotic cardiovascular diseases among patients with hypertension and diabetes attending Irrua Specialist Teaching Hospital (ISTH), Irrua, Nigeria.

## Materials and methods

### Study design, location and population

The study was a descriptive, cross-sectional study conducted at the General Out Patient Department (GOPD) of Irrua Specialist Teaching Hospital (ISTH), a teaching hospital in Irrua, Edo State, Nigeria. Adults aged 18 years and above who had hypertension or diabetes presenting to the GOPD comprised the study population. These included those newly diagnosed as well as those with established diseases. However, pregnant women and those critically ill who were too weak to participate as well as those requiring hospitalisation were excluded from the study.

### Sample size determination and sampling

Sample size was calculated using fisher’s formula and a prevalence of 37% of cardiovascular disease reported by Adebayo in south western Nigeria [6] to be 358. With an anticipated 10% attrition, 394 patients presenting to the GOPD who met the above selection criteria and gave written informed consent were selected using systematic random sampling. About 400 patients with hypertension or diabetics are seen monthly in the GOPD from hospital records. The sampling frame was determined to be 1200 for the three months the study lasted. This gave a sample interval of 3. The first patient for the day was selected by balloting. Then every third patient was then selected until the required sample size was achieved.

### Data collection

The Cardiovascular risk assessment was done using the Framingham risk score and entered into an interviewer administered structured questionnaire (using the Framingham 10year Risk of General Cardiovascular Disease*)*. The questionnaire included sociodemographic characteristics of respondents, their medical history including history of hypertension and diabetes. It also included clinical assessment of patients. Anthropometric measurements including weight (Kg), height (m), waist circumference (m), from which body mass index (BMI) and waist height ratio (WHtR) were determined and recorded. Omron 6 digital sphygmomanometer (OMRON M2 Classic Intellisense) was used to measure BP [10]. An adult sized cuff was applied on the mid arm used with patient sitting down and resting his/her back and arm exposed. Three measurements were taken 10 minutes apart and the average of the last two was determined and recorded. Blood samples were collected for Lipid Profile, Fasting Blood Glucose, and Glycosylated Haemoglobin. The patients on recruitment were asked to come the following morning before eating for sample collection. Venous blood was collected with a 10ml syringe from the superficial veins of the forearm with the aid of a tourniquet. Blood for lipid profile was placed in a lithium heparin sample bottle while blood for fasting blood glucose and glycosylated haemoglobin were both placed in a fluoride oxalate bottle and transported to the chemical pathology laboratory of the hospital where they were analysed with the assistance of a Chemical Pathologist using enzymatic colourimetric method. The information and results obtained were entered into the questionnaire which was pretested in the nearby general hospital prior to the commencement of the study.

The Framingham Risk Score was determined using the following parameters from the patients: age, sex, total cholesterol, HDL-cholesterol, systolic blood pressure, and smoking habits [11]. Ten year CVD risk was calculated in percentage using an online calculator (mdcalc.com) and patients were categorized as low risk (< 10%), intermediate risk (10–20%), and high risk (> 20%).

Physical activity was determined using the WHO criteria of at least 30 minutes moderate to intense physical activity (like brisk walking, jogging, running, swimming, cycling and skipping) daily for a minimum of 5 days a week or a cumulative 150 minutes of moderate to intense physical activity per week [12]. Patients were asked whether they engaged in physical activity. For those who did, they were further asked the type of physical activity they engaged in and the duration per session as well as the number of sessions per week. Thus, patients with less than 150 minutes of moderate to intense physical activity per week were categorized as being inactive while those with at least 150 minutes of moderate to intense physical activity were categorized as active.

Respondents were weighed using a digital weighing scale (Secca 770 Floor Digital Scale, Hamburg Germany) to the nearest 0.1 kg, standing bare footed and on light clothing. Their height was measured with a stadiometer (Secca 240 wall mounted, Hamburg Germany) to the nearest 0.1 cm with their feet bare and without cap or head gear. The waist circumference of study participants was measured at the midpoint between the lowest rib and the iliac crest to the nearest 0.1 cm with a non-stretchable tape. Obesity was evaluated using both Body Mass Index (BMI) and Waist-Height Ratio (WHtR) which were calculated by dividing the weight (kg) by the square of the height (m^2^) and dividing the waist measurement by the height respectively [13]. BMI was categorised into Underweight (<18kg/m^2^), Normal Weight (18–24.9 kg/m^2^), Overweight (25–29.9 kg/m^2^), and Obese (≥30 kg/m^2^) [14, 15]. WHtR was categorized into obese (≥ 0.5) and not obese (< 0.5) as recommended [14].

### Data analysis

All data collected were entered in excel spreadsheet and analysed using Stata version 16 (Statacorp LLC, Texas, USA). Categorical variables were summarized using proportions, frequency and percentages. Continues variables were presented as mean, and standard deviations. Bivariate associations were analysed using chi square test for categorical variables while students t-test was used for continuous variables. Binary logistic regression analysis was used to obtain the association between the outcome and physical activity/obesity and the other independent variables. The dependent variable (outcome) was the level of risk of CVD, as determined by the Framingham 10-year Risk of General Cardiovascular Disease. For analysis purpose, the risk levels were re-categorised into two with low and intermediate risk as 1 category while high risk was category 2. In all, the level of significance was set at p = 0.05.

### Ethical clearance

Approval for this study was sought and obtained from the Ethical and Research Committee of ISTH (ISTH/HREC/20221204/280) and informed written consent obtained from the participants prior to the commencement of study and recruitment of subjects respectively. The study was conducted between 1^st^ May 2023 and 31^st^ July 2023.

## Results

There were 412 patients that were approached and screened, 7 were found not eligible based on the selection criteria, 5 eligible patients declined to participate and 6 patients had incomplete data. Of the 394 patients that participated in the study and their data analysed, 39.1% of them were aged 60years and above, 39.8% were 40 to 49 years of age and the remaining 21.1% were aged 18 to 39 years., A total of 55.3% of respondents were females, 46.2% were nongovernment employees and 44.6% had tertiary level of education. 70.3% of the patients were not physically active. 42.4% of patients were overweight and 34.0% obese when assessed with BMI, but with WHtR, 75.1% of respondents were obese. 41.8% of respondents had a high risk of developing CVD in 10 years using Framingham categorisation while 37.6% and 20.6% of respondents had low and intermediate risk respectively. 259 (65.7%) respondents had hypertension while 205 (52.0%) had diabetes. (Table 1).

**Table 1:** Sociodemographic characteristics of respondents (N = 394).

Variables	Frequency	Percent (%)
**Age (years)**		
< 40	83	21.1
40–59	157	39.8
≥ 60	154	39.1
**Sex**		
Female	218	55.3
Male	176	44.7
**Occupation**		
Non-Government Employee	182	46.2
Government Employee	129	32.7
Unemployed	83	21.1
**Level of Education**		
Below Secondary	109	27.7
Secondary	109	27.7
Tertiary	176	44.6
**10 Year CVD Risk**		
Low Risk	148	37.6
Intermediate Risk	81	20.6
High Risk	165	41.8
**BMI**		
Underweight	6	1.5
Normal	87	22.1
Overweight	167	42.4
Obesity	134	34.0
**Waist Height Ratio**		
Non Obese (<0.5)	98	24.9
Obese (≥0.5)	296	75.1
**Physical Activities**		
No	277	70.3
Yes	117	29.7
**HTN**		
Yes	259	65.7
No	135	34.3
**DM**		
Yes	205	52.0
No	189	48.0

The mean parameters of the respondents are as shown in Tables 2 and 3. Respondents had a mean age of 54± 15.47 years, a mean BMI of 28.08±4.59kg/m^2^ and a mean WHtR of 0.58±0.04. The mean systolic and diastolic BP were 134.37±22.97mmHg and 85.08±19.09mmHg respectively. They also had a mean FBS of 103.54±33mg/dl. The mean glycated haemoglobin was 10.44±1.54%. Female respondents had significantly lower mean age (p<0.001), SBP (p = 0.009), and DBP (p<0.004). They were however more obese with a significantly higher mean BMI (p = 0.011) and WHtR (p<0.001). There was no significant difference in the mean FBS (p = 0.725) and HbA1C (p = 0.524) among both sexes (Table 2).

**Table 2:** Comparison of parameters of respondents based on gender (N = 394).

Variables	Mean ± SD			95% Confidence Interval	t-test	P value
	Total Population (N = 394)	Male (n = 176)	Female (n = 218)			
Age (years)	54 ± 15.47	58±14.74	51±15.39	-10.01 to -3.99	-4.572	< 0.0001*
Body mass index (kg/m^2^)	28.08 ± 4.59	27.43±3.85	28.61±5.07	0.27 to 2.09	2.550	0.011*
Waist Height Ratio	0.58 ± 0.04	0.57±0.04	0.59±0.04	0.01 to 0.03	4.934	< 0.0001*
Systolic Blood Pressure (mmHg)	134.37 ± 22.97	137.74±22.88	131.65±22.74	-10.63 to -1.55	-2.636	0.009*
Diastolic Blood Pressure (mmHg)	85.08 ± 19.09	88.21±19.88	82.57±19.10	-9.52 to -1.76	-2.861	0.004*
Fasting Blood Sugar (mg/dl)	103.54 ± 33.53	102.88±34.50	104.08±32.80	-5.49 to 7.89	0.353	0.725
HbA1C	10.44 ± 1.54	10.39±1.57	10.49±1.53	-0.21 to 0.41	0.637	0.524

**Table 3:** Comparison of parameters of respondents with hypertension and diabetes (N = 394).

Variables	Mean ± SD					
	Total Population (N = 394)	HTN (n = 259)	DM (n = 205)	95% Confidence Interval	t-test	P value
Age (years)	54 ± 15.47	59±14.55	52±15.77	-9.77 to -4.23	-4.959	<0.0001
Body mass index (kg/m^2^)	28.08 ± 4.59	28.13±4.85	27.77±4.32	-1.21 to 0.49	-0.833	0.405
Waist Height Ratio	0.58 ± 0.04	0.58±0.04	0.58±0.04	-0.01 to 0.01	0.000	1.000
Systolic Blood Pressure (mmHg)	134.37 ± 22.97	144.49±20.99	126.93±23.45	-21.62 to -13.50	-8.496	<0.0001
Diastolic Blood Pressure (mmHg)	85.08 ± 19.09	93.19±17.64	78.44±19.86	-18.18 to -11.32	-8.459	<0.0001
Fasting Blood Sugar (mg/dl)	103.54 ± 33.53	103.96±33.78	113.60±42.10	2.72 to 16.56	2.737	0.006
HbA1C	10.44 ± 1.54	10.47±1.55	10.26±1.55	-0.49 to 0.07	-1.449	0.148

Respondents with hypertension had a significantly higher mean age (p<0.001), SBP ((p<0.001), and DBP (p<0.001) compared to those with diabetes. They however had a significantly lower FBS (p = 0.006). There was no statistically significant difference in the mean BMI (p = 0.405) and HbA1C (p = 0.148) among hypertensive and diabetic patients in the study (Table 3).

Table 4 shows the association between obesity, physical activity, other factors and the risk level for cardiovascular disease among respondents. The risk level of CVD was significantly higher in patients who were overweight and obese as measured by their BMI (p<0.001) compared with those who had normal weight or were underweight with the risk level of CVD increasing with increase in BMI. Also patients who were obese as measured by a high WHtR had a significantly higher risk level for CVD compared to those with normal WHtR (p<0.001). Also, patients who were not physically active had a significantly higher risk level of CVD (P<0.001) compared to those who were physically active with the risk level of CVD reducing with increase in physical activity. Being hypertensive (p<0.001) or diabetic (p<0.0001) was associated with high CVD risk. Also female respondents had a significantly higher risk level for CVD compared to male respondents (p<0.001).

**Table 4:** The association between obesity, physical activity, other factors and the risk level for cardiovascular disease.

BMI	Low risk	Intermediate risk	High risk	P-Value
Underweight	3 (50.00)	2 (33.33)	1 (16.67)	< 0.01
Normal	49 (56.32)	22 (25.29)	16 (18.39)	
Overweight	63 (37.73)	32 (19.16)	72 (43.11)	
Obesity	33 (24.63)	25 (18.65)	76 (56.72)	
**Waist Height Ratio**				
Non obese	62 (63.26)	23 (23.47)	13 (13.27)	< 0.01
Obese	86 (29.05)	58 (19.60)	152 (51.35)	
**Physical Activities**				
No	75 (27.08)	69 (24.91)	133 (48.01)	< 0.01
Yes	73 (62.39)	12 (10.26)	32 (27.35)	
**HTN**				
Yes	63 (24.3)	67 (25.9)	129 (49.8)	< 0.01
No	85 (63.0)	14 (10.4)	36 (26.7)	
**DM**				
Yes	68 (33.2)	35 (17.1)	102 (49.8)	< 0.01
No	80 (42.3)	46 (24.3)	63 (33.3)	
**Sex**				
Female	66 (30.3)	46 (21.1)	106 (48.6)	< 0.01
Male	82 (46.6)	35 (19.9)	59 (33.5)	

High cardiovascular risk was observed to occur 2.5 and 3.2 times in participants with peripheral and central obesity respectively compared to those with low cardiovascular risk (p<0.001; OR 2.52; CI: 1.64–3.86) and (p<0.001; OR 3.23; CI: 2.01–5.19) respectively. Respondents who were at high risk level for CVD were 2.5 times likely to be physically inactive compared to those at lower risk level (p<0.001; OR 2.45; CI: 1.53–3.92). Respondents who were at high risk level for CVD were 2.7 and 2.0 times likely to have hypertension (p<0.001; OR 2.73; CI: 1.74–4.29) and diabetes (p<0.001; OR 1.98; CI: 1.32–2.98) respectively compared to those at low risk level. High risk level for CVD was 2.1 likely to be seen in Females (p<0.001; OR 2.06; CI: 1.36–3.11) compared to low risk levels (Table 5).

**Table 5:** Logistic regression analysis of association between Physical activity, obesity and risk level for cardiovascular disease.

Variables	OR	95% Confidence Interval	P-Value
**BMI**			
Not Obese	1	Ref	
Obese	2.52	1.64–3.86	<0.01
**WHtR**			
Not Obese	1	Ref	
Obese	3.23	2.01–5.19	<0.01
**Physical Exercise**			
Yes	1	Ref	
No	2.45	1.53–3.92	<0.01
**HTN**			
No	1	Ref	
Yes	2.73	1.74–4.29	< 0.01
**DM**			
No	1	Ref	
Yes	1.98	1.32–2.98	< 0.01
**Sex**			
Male	1	Ref	
Female	2.06	1.36–3.11	< 0.01

OR = Odds ratio, Ref = Reference, p<0.05 was statistically significance.

## Discussion

Non-communicable diseases (NCDs), such as diabetes mellitus and cardiovascular disorders, have been recognised by the World Health Organisation as posing a serious threat to economies and society [16]. About 73% of all deaths worldwide in 2017 were due to NCDs, and 28.8 million of those fatalities were linked to risk factors such as high blood pressure, high blood sugar, or a high body mass index (BMI) [17]. Studies had shown 10-year cardiovascular disease risk assessment to be highly predictive [18, 19], yet this is not routinely done in our clinical practice by physicians [20]. In this descriptive, cross-sectional study, physical activity and obesity were independently associated with increased risk of cardiovascular disease. Heart disease can result from insufficient physical activity. Additionally, it can raise the likelihood of developing other diseases that carry risk factors, such as diabetes, obesity, high blood pressure, and high cholesterol levels. Chances of developing heart disease can be lowered by regular exercise. Undoubtedly, physical activity improves the energy expenditure of an individual, which can help them maintain energy balance or even lose weight if they don’t overeat to compensate for the extra calories they burn. Consequently, physical activity reduces total excess body fat, including waist fat and total body fat, slowing the progression of central obesity [15, 21–23].

The majority of the study participants were not physically active, overweight/obese and at risk of developing CVD. High cardiovascular risk was observed to occur 2.5 and 3.2 times in participants with peripheral and central obesity respectively compared to those with low cardiovascular risk in those who were not obese. Studies have reported that obesity leads to the development of CVD and CVD mortality independently of other CVD risk factors [24]. Central obesity, has been found to be a CVD risk marker that is independent of BMI [25]. Not surprising, subjects with central obesity had 3.2 times higher risk level for CVD compared to those with elevated BMI with 2.5 times greater risk. This was further aggravated by inactivity.

Our study found individuals who were not physically active to have a 2.5 times greater risk level for CVD in comparison to those who engaged in physical activity. The findings are similar to that of Anjajo and colleagues who also found that diabetics who did not exercise at a moderate level had a roughly 2.4-fold increased risk of getting hypertension. The study also found moderate-intensity exercise to be substantially linked to a decreased risk of hypertension [26]. This also compares to findings of a study in an urban Chinese population where the prevalence of hypertension was inversely connected with physical activity level [27].

More than half of the respondents, in our study, were females. Females were more obese in the study compared to males as they had a higher mean BMI and WHtR. They also had a higher mean FBS and HbA1C, though they had a lower mean SBP and DBP. They also had a significantly higher risk level for CVD compared to males with a 2.06 fold increase in the likelihood of CVD risk. In conformity with our findings, publications in the area of gender and health have established that gender differences exist concerning decision-making regarding the appropriate type of treatment [28, 29].

Up to 37.2% of respondents were civil servants and as many as 21.1% were unemployed with 70.3% of the respondents physically inactive. Inactivity potentiates unhealthy weight gain and sedentary behaviour promotes it as well. Sedentary lifestyle, unhealthy diet and consequent obesity markedly increase risk of CVD [24]. As many as 72.3% of respondents had educational attainment above secondary level. Higher educational attainment may have resulted in exposure to sedentary behavioural practices leading to obesity with the resulting higher risk level for CVD in this study group.

Low levels of physical exercise and a sedentary lifestyle are widespread in many developing and developed nations. Numerous cardiovascular risk factors, such as high blood pressure, high total cholesterol, a high body mass index, and obesity are linked to this type of lifestyle as well as reduced HDL cholesterol levels [21]. One of the main public health issues is physical inactivity. It is reported that one out of every four adults worldwide faces the issue of inactivity [22]. An earlier study revealed that one-third of adult South Africans consider sedentary behaviour as the norm [23].

Clients in this facility can have the benefit of lifestyle changes if referrals to specific related units is intensified: dietetics, physical therapy unit as well as behavioural and lifestyle medicine clinics which are operational. Their inputs will significantly benefit clients in this locality with such significant ASCVD risk. Also public enlightenment on healthy lifestyle habits like increased physical activity and weight reduction practices will reduce ASCVD risks in the general population leading to a healthier society.

## Limitations

The study did not evaluate the income level of patients. This, together with the educational status and employment type would have given a better assessment of the socioeconomic status of respondents.

The study was a cross-sectional study and therefore, a cause–effect relationship could not be established among variables.

The study being a hospital-based study did not take into cognizance of patients attending other health facilities or alternative care including those on self-medications.

## Conclusion

In conclusion, the study found majority of patients with hypertension and diabetes attending ISTH to be obese and physically inactive. The study also found a significant relationship between unhealthy lifestyle (physical inactivity and obesity) and the risk of atherosclerotic cardiovascular diseases among these patients. Lifestyle modifications, including increased physical activity and weight management, play a critical role in reducing the risk of ASCVD in this high-risk population and should be incorporated into patient management. Comprehensive care, risk assessment, and patient education are essential components of managing these interconnected risk factors and improving cardiovascular outcomes.

Findings from this study would help improve patient outcomes through the development and implementation of policies and practice that will help patients engage in regular exercise, weight reduction, smoking cessation and alcohol reduction/cessation. This should be given top priority at all levels of healthcare. Health education as well as other measures that will help the general public engage in more physical activity as well as weight reduction should be instituted by the government and other relevant stakeholders.

## Supporting information

S1 File Results of data collected attached as supporting information. (XLS)
